# Deep learning-enabled Inference of 3D molecular absorption distribution of biological cells from IR spectra

**DOI:** 10.1038/s42004-022-00792-3

**Published:** 2022-12-22

**Authors:** Eirik Almklov Magnussen, Boris Zimmermann, Uladzislau Blazhko, Simona Dzurendova, Benjamin Dupuy–Galet, Dana Byrtusova, Florian Muthreich, Valeria Tafintseva, Kristian Hovde Liland, Kristin Tøndel, Volha Shapaval, Achim Kohler

**Affiliations:** 1 Faculty of Science and Technology, Norwegian University of Life Sciences, Ås, Norway; 2 Department of Biological Sciences, University of Bergen, Bergen, Norway

**Keywords:** Infrared spectroscopy, Imaging techniques, Cheminformatics

## Abstract

Infrared spectroscopy delivers abundant information about the chemical composition, as well as the structural and optical properties of intact samples in a non-destructive manner. We present a deep convolutional neural network which exploits all of this information and solves full-wave inverse scattering problems and thereby obtains the 3D optical, structural and chemical properties from infrared spectroscopic measurements of intact micro-samples. The proposed model encodes scatter-distorted infrared spectra and infers the distribution of the complex refractive index function of concentrically spherical samples, such as many biological cells. The approach delivers simultaneously the molecular absorption, sample morphology and effective refractive index in both the cell wall and interior from a single measured spectrum. The model is trained on simulated scatter-distorted spectra, where absorption in the distinct layers is simulated and the scatter-distorted spectra are estimated by analytic solutions of Maxwell’s equations for samples of different sizes. This allows for essentially real-time deep learning-enabled infrared diffraction micro-tomography, for a large subset of biological cells.

## Abstract (web summary)

Infrared spectroscopy-based diffraction tomography has a high potential to be used for 3D reconstruction of intact samples, however, the inverse problem is highly non-linear and remains challenging. Here, the authors solve full-wave inverse scattering problems using deep convolutional neural networks and perform 3D spectral reconstruction by diffraction tomography from scatter-distorted IR spectra.

## Introduction

Infrared (IR) micro-spectroscopy is a versatile and widely used technique for studying the chemistry of biological cells and tissues. The chemical composition is estimated by the amount of attenuated radiation as light propagates through samples mounted on a microscopic stage^1,2^.

Molecular bonds in the samples vibrate and absorb radiation in quantities proportional to their concentration, attenuation coefficient and optical path length, according to Beer–Lambert’s law. IR spectra can thus provide a succinct fingerprint of the chemical components in the samples.

It is however well known that measured spectra of intact samples generally contain strong contributions from light scattering, which severely distorts the idealized scenario where the measured absorbance peaks are proportional to the concentration of absorbing molecular bonds, described by Beer–Lambert’s law^3^. The loss of radiation due to scattering is determined by the sample’s complex refractive index function which depends on the sample morphology, the distribution of the effective refractive index and molecular absorption. Thus, light scattering generally mixes the molecular and physical information in a very intricate way, since the scattering itself depends also on molecular absorption.

Gustav Mie solved Maxwell’s equations analytically and estimated the light scattering on perfect spheres at the beginning of the twentieth century. Analytical solutions for other ideal morphologies, such as cylinders, slabs and concentric spheres have been developed later^4,5^. Mie-type scattering is particularly pronounced and highly non-linear when the radius of the sample is on the same order of magnitude as the wavelength of the radiation. Since both biological cells and the wavelength of IR radiation are on a micrometer scale, non-linear Mie-scattering effects such as wiggles and ripples are ubiquitous in biological applications of IR micro-spectroscopy. Wiggles are broad oscillations largely due to the interference of the undisturbed and disturbed wave passing through the sample, and ripples are sharp oscillations in the spectra arising from standing waves^4–7^.

Since IR spectroscopy generally is employed to study the chemical information in measured spectra, it has been considered imperative to correct the spectra by removing all non-chemical, scattering contributions to the spectral signals. Several Extended Multiplicative Signal Correction (EMSC) based approaches^8–10^ are widely applied to pre-process measured spectra and estimate the pure molecular absorption spectra.

The state-of-the-art correction algorithm is the so-called Mie Extinction EMSC^11^ which has been shown to efficiently remove Mie-scattering from IR spectra. Deep learning models trained on spectra corrected with EMSC are also being applied for Mie-scattering correction in IR spectroscopy of biological samples and have been shown to outperform EMSC both in terms of speed and precision, in particular for imaging data^12^. Additionally, there have been attempts to train models on data sets of purely simulated scatter-distorted spectra for non-biological samples^13^.

Research and applications of IR spectroscopy have mainly either considered it a label-free technique or focused on extracting and interpreting the molecular absorption signals in measured spectra. Although positions and shapes of ripples and wiggles have been shown to be highly diagnostic for optical and morphological properties of micro-samples^7,14^, exploitation of the information inherent in the scattering signals for biological samples is mostly uncharted territory. We hypothesize that measured spectra of biological micro-samples contain sufficient information, both chemical and physical, to infer the morphology and the effective refractive index distribution of the samples as well as the spatially resolved chemical composition, and we suggest exploiting the information contained in the scattering rather than correcting for it.

IR spectroscopic instruments can very quickly collect spectra either in single-element mode (1D) or in imaging mode (2D) using, e.g. a focal plane array (FPA) detector. Whereas the acquisition of 1D and 2D spectral information is straightforward in state-of-the-art IR microscopes, obtaining 3D spectral information is experimentally challenging. IR spectro micro-tomographic reconstruction has been experimentally realized in ref. ^15^ where a computed tomography approach was applied to obtain the 3D distribution of the chemical composition of an intact sample. The approach requires rotating the sample and acquiring several two-dimensional projection images using a custom-made sample holder. In addition to being time-consuming, the method is fairly challenging to employ since the sample must be very precisely rotated, and biological samples can be difficult to keep in focus and stable throughout the rotation. Furthermore, light scattering can influence the collected spectra and generally has to be taken into account in the computed micro-tomography. This will generally entail the necessity of performing scatter-correction before the 3D rendering, which is highly non-trivial for such tomographic sections.

In addition, the accuracy with which spatial distribution of the molecular absorption can be resolved is limited by diffraction and even further reduced for highly scattering samples, since for every voxel light scatters into the detector from neighboring voxels. Light scattering is also an issue with conventional 2D FPA imaging. Since scattering is intimately linked to absorption, nearby pixels in IR images are influencing the absorbance signal recorded at them, and this is reducing the resolution with which one can distinguish molecular information in different parts of the sample drastically. In for example biological cells with fairly thin cell walls, it can thus be hard to clearly distinguish the chemical composition of the cell wall from that of the rest of the cell. This is something which cannot be ameliorated by any known scatter-correction method.

This beckons us to consider novel routes to obtaining molecular and structural information in micro-samples and consider if the resolution can be improved by explicitly exploiting the scattering information in the measured spectra, thereby obtaining the molecular absorption sharply distinguished in different regions of the sample.

To this end, we consider a deep learning-based inverse scattering solver for volumetric molecular absorption reconstruction, where we reconstruct the 3D complex refractive index by making use of the information-rich scattered field of micro-particles probed by conventional IR microscopes. Deep convolutional neural networks (DCNNs) have previously been shown to be able to separate chemical and physical information in IR spectra^12^. We, therefore, opt for performing 3D spectral reconstruction by employing DCNNs. This approach would be significantly faster than the computed tomography approach, as the collection of the spectra is done in a matter of seconds and our DCNNs infer the distribution of chemical components essentially in real-time. Additionally, in our proposed approach the scattering does not need to be corrected separately, since our approach is actively taking advantage of the light scattering and thus making tedious scatter-correction superfluous.

Inferring the 3D complex refractive index function from measured IR spectroscopic data is an electromagnetic inverse scattering problem (ISP). A plethora of methods for solving inverse problems in electromagnetics exists^16–18^. However, most methods are based on iterative algorithms that are computationally expensive or only good approximations for weakly scattering samples and not applicable to IR spectroscopy. There have been attempts at exploiting DCNNs for solving ISPs in the field of photonics, where the DCNNs were used to predict parameters of photonic systems such as the thickness of multilayered thin films^19^ and multilayer nano-particles^20^. However, such ISPs are comparatively easy to solve since the solution space is fairly small and consists of merely a few parameters describing the photonic system. In refs. ^21,22^, a morphologically more complex photonic system was considered, where they solved a full-wave ISP using DCNNs. However, they only considered electromagnetic radiation with frequencies significantly lower than that of IR radiation and they also assumed non-absorbing samples. This approach would therefore also not be directly applicable to solving ISPs modeling IR spectroscopy.

For the case of IR spectroscopy of cells and tissues we have scatters of arbitrary morphologies, and additionally, we are obliged to consider the molecular absorption and the optical properties of the instrument, which greatly increases the complexity of the ISP. Generally, ISPs are ill-posed, meaning that the complex refractive index function cannot be uniquely determined by the scattered electromagnetic fields. However, in the case of IR spectroscopy, molecular absorption spectra are highly collinear, since most molecules consist of some of the same vibrating chemical bonds. Thus, if we condition the ISP to only take molecular absorption spectra as found in nature into account, we can largely handle the ill-posedness.

In this work, we demonstrate that it is feasible to use a single IR spectroscopic measurements to obtain the 3D optical and structural properties of biological samples, as well as find the molecular absorption in spatially resolved regions of the sample. In particular, we retrieve the molecular absorption of both the cell wall and interior of intact biological cells from single IR spectra. We show that we can obtain the entire 3D distribution of the complex refractive index under some assumptions, and demonstrate this for two-layered, concentrically spherical samples in simulations and experiments. This approach is generally applicable for samples of arbitrary chemical composition, optical properties and morphology, assuming one can simulate the necessary training data. We demonstrate the approach for the case of samples which can be well approximated by a model of concentric spheres, which is the case for very many biological and non-biologic samples, such as algae, bacteria, several animal and plant cells, pollen and microplastics to mention some. By validating the results with experimental data, we show that our model does indeed work well for biological cells with chemically and optically distinct cell walls and interiors.

## Results

### Simulated spectral data

**Fig. 1:**
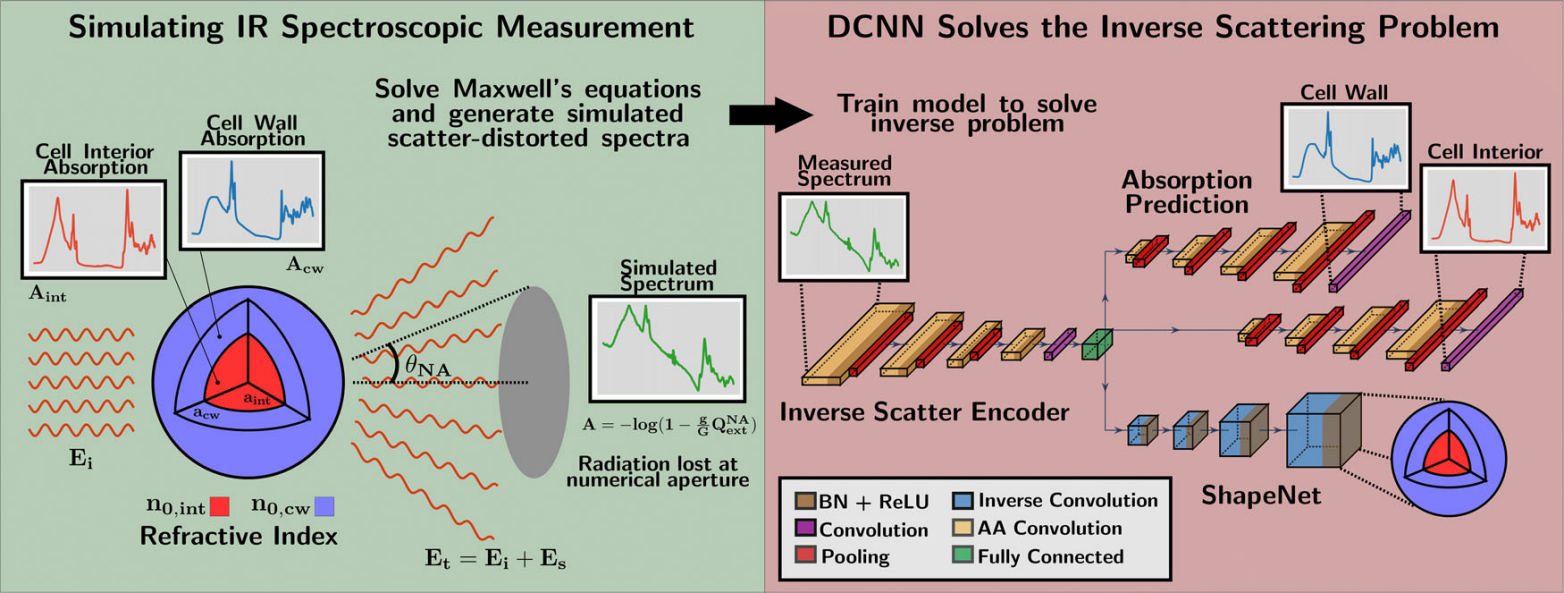
The approach for training the ISP-solver on simulated spectral data. Illustration of the proposed approach where we first solve the forward problem by exact simulation and then train a DCCN to solve the inverse problem. We start by solving Maxwell’s equations for light propagating through the sample and thereby simulating the spectroscopic measurement. We assume a two-layered concentrically spherical sample, with distinct molecular absorption and effective refractive index in the cell wall and interior. Radiation which scatters into the numerical aperture, characterized by its angle *θ*_*NA*_, is collected at the detector and contributes to the measured spectrum. These simulated spectral data are then used as training data for our DCCN, which is trained to solve the inverse scattering problem. The model is a DCNN consisting of a 1D attention-augmented CNN (InverseScatterEncoder) which decomposes the measured spectra, two 1D attention-augmented CNN with unpooling layers which infer the molecular absorption spectra of the cell wall and cell interior and a 3D transpose-CNN (ShapeNet) inferring the optical and morphological properties of the sample.

We train the model on simulated scatter-distorted spectra of samples with radii in the range *a* ∈ [1.25 and 20 μm] and refractive indices *n*_0_ ∈ [1.3, 1.6]. The outline of our model and the setup for training is seen in Fig. 1. The molecular absorption was sampled from a continuous distribution of PCA decomposed measured scatter-free IR spectra, where we used approximately 1900 spectra from 12 different data sets of different sizes.

**Fig. 2:**
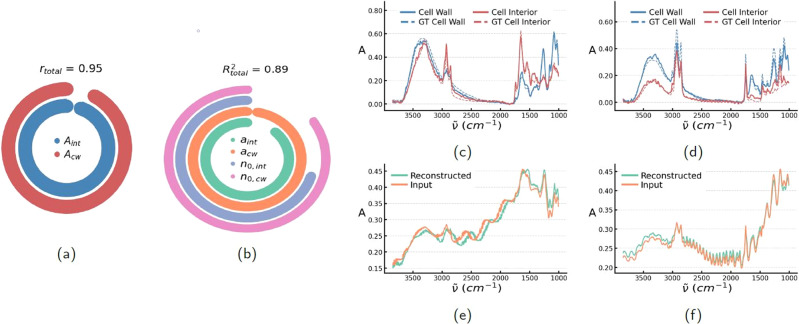
The ISP-solver applied to simulated spectral data. **a** The performance metrics for the prediction of molecular absorption, given as the Pearson correlation coefficient *r*. **b** The prediction of optical parameters quantified by the coefficient of determination *R*^2^. For both metrics the circle bar starts at 0 and a fully drawn circle implies the metric being 1. **c**, **d** The molecular absorption of the cell interior and the cell wall of the concentrically spherical sample. The dashed lines represent the ground truth and the fully drawn model’s predictions. **e**, **f** The simulated scatter-distorted spectra and the reconstructed spectra from the predicted complex refractive index functions. All metrics and spectra in **a**–**f** are coming from the continuous simulated data set which was also used for training the model. The statistical measures in **a**, **b** are estimated on a population of *n* = 10,000 simulated samples.

We see in Fig. 2, that the model is able to solve the ISP and can predict the radius, cell wall thickness and refractive indices with good accuracy, and simultaneously infer the molecular absorption of both the cell interior and cell wall. We quantify the performance of the molecular absorption prediction by the Pearson correlation coefficient *r* ∈ [0, 1], and optical and structural parameters by the coefficient of determination *R*^2^ ∈ [−1, 1]. The presented metrics are computed as an average of over 10,000 simulated scatter-distorted spectra. We see from Fig. 2b that the radius of the sample itself can be exceptionally accurately determined with ${R}_{{a}_{{{\mbox{cw}}}}}^{2}=0.99$ and also the radius of the cell interior is fairly well estimated with ${R}_{{a}_{{{\mbox{int}}}}}^{2}=0.92$. The refractive indices can also be determined reasonably accurately, but with a slightly larger uncertainty with ${R}_{{n}_{0,{{\mbox{int}}}}}^{2}=0.78$ and ${R}_{{n}_{0,{{\mbox{cw}}}}}^{2}=0.88$, respectively.

We use the model’s predictions of the molecular absorption spectra and morphological and optical parameters, and simulate the scatter-distorted spectrum from the predicted absorption and parameters, using the analytic solutions to Maxwell’s equations. This should then be very similar to the scatter-distorted spectrum which the network had as input, if the network’s predictions are accurate. We see in Fig. 2e–f that this works fairly well.

Since the same analytical solutions are used for the simulated training data and the test data, the result shows mainly the uniqueness of the solution to the inverse problem. We can conclude that we were able to achieve a high uniqueness of the problem by conditioning the problem with respect to the chemical variability employed and the layered analytical close-form solutions used.

### Fungal cells

We consider a data set containing FPA hyperspectral images of spherical cells of the *Mucor circinelloides* fungal strain. In order to have one spectrum per spherical fungal cell and emulate a single-element setup, we use binning in the image over the region covering the sample of interest. This is necessary for the application of our model to FPA data.

**Fig. 3:**
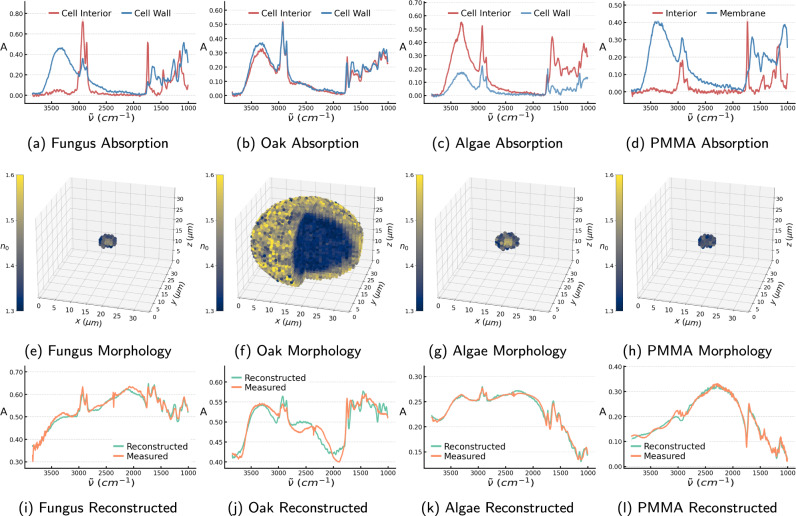
The ISP-solver was applied to measure the spectra of four different micro-samples. **a**–**d** Model predictions of the molecular absorption in the cell wall and the cell interior in samples from fungal cell, oak pollen grain, algal cell, and PMMA sphere. **e**–**h** The model’s predictions of morphology and distribution of effective refractive index in the samples. **i**–**l** Reconstructed spectrum made using the predicted molecular absorption as well as the predicted morphology and refractive indices as input to analytic solutions of Maxwell’s equations describing the scattering on concentric spheres. If the predictions are correct, the reconstruction should be very similar to the measured spectrum.

Results of application to samples of fungal cells in Fig. 3a show the predicted molecular absorption of the cell wall and the cell interior, and we see in Fig. 3i that we can very well reconstruct the measured spectra from the predicted molecular spectra and optical parameters, which corroborates the model’s predictions.

We see clearly that the model predicts molecular absorption indicative of lipids (triglycerides) for the cell interior with pronounced peaks at 1160, 1465, 1745, and around 2900 cm^−1^. It also predicts the cell wall to be rich in proteins and carbohydrates as seen from the strong peaks around 1680 − 1530 cm^−1^ and 1200 − 1000 cm^−1^, respectively. This has been reported in the literature and is in accordance with the expectation that the cell wall consists largely of polysaccharides such as chitin and chitosan, proteins and polyphosphates and that lipid bodies accumulate and grow in the interior of the fungal cells, which thus consists mainly of triglycerides^23^.

**Fig. 4:**
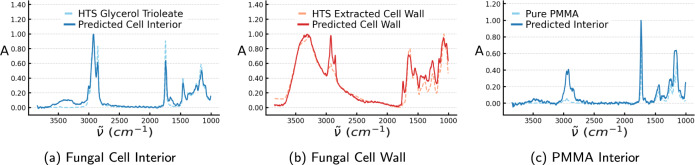
Predictions of spatially resolved absorbance compared with pure spectra. **a** The model’s median prediction of molecular absorption in the interior of the cell and HTS-FTIR measurement of the glyceryl trioleate which is known to be produced by *Mucor circinelloides*^24^. **b** Median prediction of cell wall absorption and measured HTS-FTIR spectra of the cell wall of *Mucor circinelloides* fungal cells obtained from sonication and extraction to remove the fatty acids from the total biomass. **c** The median prediction of the interior absorption of PMMA spheres and pure, scatter-free measured absorption spectrum of a PMMA sample.

In Fig. 4b we compare the median predicted molecular absorption in the cell wall with HTS-FTIR spectra of extracted cell wall from *Mucor circinelloides* strains cultivated under equivalent growth conditions as the samples used for inference by the model. The cell wall was extracted by physical disruption of the cells and then separating out the lipids by centrifugation. The remaining biomass is then generally considered to be the cell wall of such fungal cells. However, although this method removes all of the lipids it is not the case that there can be no lipids in the cell wall of the intact cell, and this could be the reason for the model predicting absorption signals in the lipid regions of the cell wall spectrum. Figure 4a shows that the model’s prediction of molecular absorption is very similar to pure glycerol trioleate, which is the triglyceride known to mainly be produced by *Mucor circinelloides*^24–26^. Quantitatively, we find that the average predicted cell interior spectrum has a Pearson correlation coefficient with the glycerol trioleate of *r*_int,GT_ = 0.97 and with the HTS-FTIR spectrum of an extracted cell of *r*_int,CW_ = 0.33, and the predicted cell wall spectrum correlates with glycerol trioleate with *r*_cw,GT_ = 0.35 and the extracted cell wall with *r*_cw,CW_ = 0.97.

### Algal cells, pollen grains, and PMMA samples

Additionally, we apply our approach to spectra of microalgae *Vischeria Polyphem* and spectra of both juniper and oak pollen grains which have both been desiccated, and the juniper pollen were subsequently re-hydrated to better retain their original spherical form. We see that the model predicts larger absorption in the carbohydrate region and less in the protein region in the cell wall. This is in accordance with the belief that the cell walls of both pollen grains and algae are rich in polysaccharides and consist mainly of cellulose and pectin, and in the case of pollen also sporopollenin^27,28^. Furthermore, the model predicts some absorption in the lipid regions of the spectra for the cell wall. This could be caused by a membrane of phospholipids outside the cell interior which is known to occur in some cases for such microalgae^29,30^. We see also in Fig. 3j, k that the scatter-distorted spectra can be fairly well reconstructed from the predictions for both algae and pollen. However, the pollen grain reconstruction is slightly less accurate than for the other samples as we see in Fig. 3j presumably due to the fact that the pollen grains may differ considerably in their shape from concentric spheres, since they are often rather ellipsoidal and have a spiky surface. Generally, we expect more accurate results when the samples are well approximated as concentric spheres, while stronger deviations can be expected for samples where this is not the case.

We also apply our model to spectra of polymethyl methacrylate (PMMA) spheres where we see the molecular absorption in Fig. 3d and in Fig. 3l that also this measured spectrum can very well be reconstructed from the predictions. We note that the PMMA spheres are single-layered spheres, but our model which has only been trained on two-layered spherical samples still handles this quite well as it predicts the PMMA spheres to have a very thin membrane at about 0.4 μm. The predicted membrane chemistry thus does not significantly influence the overall absorbance.

We note that both the molecular composition and the morphology of these samples are very diverse, since we have several types of biological and non-biological samples and sample sizes ranging from 2 − 3 μm to 19 − 20 μm, which demonstrates the wide applicability of the model.

### Morphological and optical predictions

**Fig. 5:**
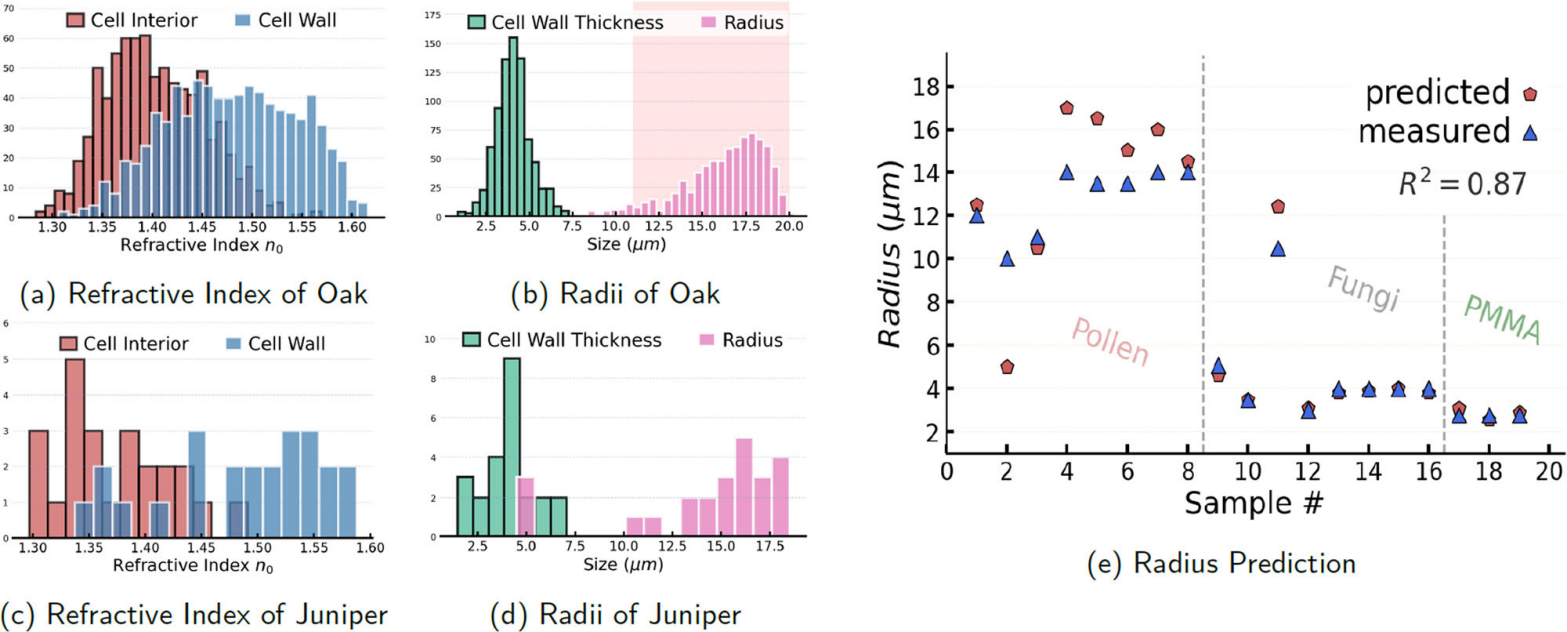
Predictions of morphology and effective refractive index. **a** Predicted refractive indices for 800 spectra of oak pollen grains. **b** The predicted sizes of the oak pollen grains and their cell wall thicknesses. The sizes given in the literature are marked with a pink background in the histogram. **c** Predicted refractive indices for 24 spectra of juniper pollen grains and in **d** their predicted sizes. **e** Comparison of the radii predicted from our model and those measured from light microscopy images. We show data from three different sample types, namely juniper pollen grains, fungal cells, and PMMA spheres.

As it is very difficult to unequivocally validate the reconstruction of the morphology and distribution of refractive index, we seek validation by looking at the predicted radii and compare them to the radii which can be fairly accurately measured from light microscopy images. Figure 5e shows that the predictions of samples’ sizes for fungi, juniper pollen and PMMA are quite accurate with a coefficient of determination at *R*^2^ = 0.87. The physical parameters are given directly by the model but could as well have been extracted from the 3D voxel map. However, it turned out that it was in practice more difficult to have a general rule for separating the layers in the continuous voxel map compared to just using a separate output for the physical parameters for the two layers. Further examples of the reconstructed shapes of three fungal cells alongside an FPA image of them for comparison can be found in Supplementary Fig. 1.

For the fungal cells, the model predicts not only the correct overall size, it also predicts the cell wall to be very thin, at around 1−2 μm, which is known to generally be the case^23^. Furthermore, the model’s median prediction of the interior of the fungal cells is *n*_0_ = 1.45 which coincides very well with the known refractive index for glycerol trioleates at ${n}_{{0}_{{{{{{\mathrm{GT}}}}}}}}=1.47$.

We see from Fig. 5b that the predicted radii for the spectra of different oak pollen species are all in the range between 11−20 μm and the cell wall is predicted to be relatively thin at 3−4 μm, which has been reported in the literature^31,32^. From Fig. 5a we also see that the grains are predicted to have an optically dense cell wall and a somewhat more translucent interior which is believed to be the case for pollen grains which generally have tough outer shells. The same holds true for the juniper pollen grains as seen in Fig. 5d.

We note again that some of these pollen grains have a slightly nonzero eccentricity and they have rough surfaces which means that they are not always perfectly spherically symmetric samples. We, therefore, see in Fig. 5e that for some of the pollen grains, the predicted radius is slightly inaccurate, which is presumably due to the fact that the shape of the samples in some cases differs considerably from a concentric sphere. In general, we expect that for samples which are not spherically symmetric, the model’s prediction accuracy may decrease. None the less our model is mostly able to make sensible inferences of the nearly spherically symmetric morphology of the sample in a useful manner, which gives testament to our model being able to find the closest solution for a non-spherical sample in the space of concentrically spherical samples.

Furthermore, the model predicts all of the PMMA samples to have a radius of 2.4−2.9 μm which coincides very well with the radius of 2.75 μm reported by the manufacturer, and it also correctly predicts that the interior’s and membrane’s refractive index of the PMMA samples were approximately equal and both at approximately *n*_0_ = 1.39−1.48, in fairly good accordance with literature^33^.

This provides further evidence that the model can predict the index of refraction for measured samples, which can be particularly useful since the refractive index is often only known for the nanometer wavelength range. With our approach, we could directly get the refractive index in the micrometer region without having to extrapolate from reference values in the nanometer region.

### Diffraction micro-tomography

We have predictions of the 3D distributions of the effective refractive index of both the pollen grains and the algal and fungal cells, seen in Fig. 3k–n, where we see that we can reconstruct the 3D distribution of optical properties and the sample morphology, from the measured spectra. This shows that it is indeed possible to solve the ISP and perform volumetric imaging based on the scattered fields collected at the detector in an IR spectrometer. That is, we have essentially performed diffraction micro-tomography for samples which are approximately two-layered concentric spheres.

## Discussion

We have demonstrated the feasibility of infrared diffraction micro-tomography for approximately radially symmetric systems, and that we, to a large degree of accuracy, can solve the full-wave Inverse Scatter Problem for IR spectroscopic data. We showed that we can obtain the molecular absorption of both the cell wall and interior from a single measured scatter-distorted spectrum of spherical PMMA samples and approximately concentrically spherical fungal and algal cells and pollen grains from juniper and oak, where we emphasize that the spectra were obtained at three different instruments and at two different laboratories. Furthermore, we have shown that we can infer the morphology and optical properties of a sample from the scattering signatures of the measured IR spectrum. That is, we can predict the full complex refractive index function from measured IR spectra, which would allow for biochemical volumetric imaging of intact cells with conventional IR spectroscopic instruments, thus we have shown that we essentially perform deep learning-enabled diffraction micro-tomography for a large subset of samples.

This allows studying the 3D distribution of chemical composition in simple intact biological samples, using only conventional, fast and easily applicable IR spectrometers. Simultaneously, we obtain the effective refractive index in the different layers of the sample. This in itself can be very useful since reference values for refractive indices in different materials are usually not given in the IR region of the electromagnetic spectrum. Our model infers the effective refractive index explicitly for IR radiation, assuming that it is constant in the wavelength region we consider, which is generally believed to be the case. Finally, this enables us to perform essentially real-time sample sizing and inference of cell wall thickness. Thus our approach is very valuable for biological and material science research and for industrial applications.

We know that we can exactly simulate the scattering of light in a single-element IR experiment to arbitrary accuracy. This is not the case for an FPA measurement, where it is not possible to estimate the scattered radiation collected at a given pixel within a reasonable time. This means that there is an inherent inaccuracy in conventional scatter-correction methods when applied to FPA images, since we know that radiation scatters from nearby pixels into the pixels which we wish to correct, and this cannot be explicitly taken into account without knowing the scattered radiation at single pixels. That is, traditional FPA scatter-correction methods can thus only remove the scattering signals, but not remove the contributions from the scattered absorption signal from nearby pixels. This leads to a blurring of the molecular absorption maps when you scatter-correct FPA images pixelwise. We thus expect our model to yield better inference of the absorption in the cell wall and interior than in the FPA image, particularly for small samples.

We note, furthermore, that in theory there is conceptually nothing stopping us from making volumetric chemical images beyond the diffraction limit, since we only need to measure the overall scattered radiation at the detector to reconstruct the individual voxels of the sample. That is, we do not have the problem of not being able to optically resolve two neighboring pixels in the image, since our approach only needs to consider the total lost radiation at the detector. The limiting factor is now the uniqueness of the solutions to the electromagnetic inverse scattering problem. This will be further addressed in future works.

In this study, we consider two-layered concentric spheres and corroborate the model for samples which are known to have two distinct layers. We note that modeling of two-layered spheres, albeit a simplification, is applicable to a large array of biological and non-biological samples, such as algae, bacteria, several animal and plant cells, pollen, micro-plastic, etc. Although modeling of two concentric layers, cannot always capture all the chemical variance within the sample, it is in many cases a good approximation, since the largest variability often exists between the cell interior and the cell wall of the sample, where we have a chemically distinct membrane encapsulating a more or less chemically homogeneous core. Thus it is generally very useful to be able to decouple the molecular and optical information in the cell wall and interior of the cell. Even in thin sections through cells, where the cell wall properties and cell interior properties could in principle be studied separately, the diffraction limit may make it difficult to achieve full decoupling. Therefore a more or less complete separation of cell wall properties and properties of the core of the cell as achieved by infrared diffraction micro-tomography is a great step forward.

The proposed approach would also seamlessly be applicable for cases where the radial symmetry is broken, if one can simulate realistic spectral data for such samples. We plan to apply the model in the near future to cylindrical samples, multilayer concentrically spherical samples and samples with off-center spherical inclusions. We could then capture the 3D distribution of the complex refractive index in measured samples with even more molecular fine structure and complex morphologies, and it would be feasible to reconstruct e.g. the exact position of the nucleus within a cell.

## Methods

### Electromagnetic scattering and absorption

The scattering of light impinging on several morphologically distinct scatterers can be put in a closed form, by solving Maxwell’s equations exactly^4,5^. The attenuation of radiation in an IR spectrometer is probed in the far-field region, where *ka* ≫ 1 and the scattered electric fields are considered transverse. In the far-field, the scattering on a small sample is unequivocally determined by the amplitude scattering matrix **S**, whose matrix elements we denote *S*_*j*_, where we have the transmitted field as a function of the incident field as

1 $$\left(\begin{array}{c}{E}_{\parallel ,s}\\ {E}_{\perp ,s}\end{array}\right)=\frac{i{e}^{ik(a-z)}}{ka}\left(\begin{array}{cc}{S}_{2}&{S}_{4}\\ {S}_{3}&{S}_{1}\end{array}\right)\left(\begin{array}{c}{E}_{\parallel ,i}\\ {E}_{\perp ,i}\end{array}\right)$$

where *E*_∥/⊥,*i*,*t*_ are the parallel and perpendicular components of the incident and transmitted field, *k* is the wavenumber and *a* the radial distance from the center of the scatterer. For the special case of radially symmetric scatterers, *S*_3_ = *S*_4_ = 0, i.e., **S** is diagonal.

All relevant measurable quantities can be extracted from the scattering amplitudes *S*_1_(*θ*, *ϕ*) and *S*_2_(*θ*, *ϕ*), where the angle *θ* is between the forward direction and the direction of scattered radiation and *ϕ* the azimuthal angle. Variables that are often associated with measurable quantities are the scattering efficiency *Q*_*sca*_, which is the radiation lost due to scattering, and extinction efficiency *Q*_*ext*_, which is the totally lost radiation^4^, which the optical theorem tells us is only dependant on the scattering amplitudes in the forward direction, assuming we are in the far-field region^5,34^.

Furthermore, assuming an optically lossless medium, we have the contribution from chemistry to the loss of radiation as *Q*_abs_ = *Q*_ext_ − *Q*_sca_, where the absorption efficiency *Q*_abs_ is the radiation lost by molecular absorption inside of the sample. In an IR measurement, radiation is collected not only in the forward direction but over a numerical aperture (NA), which compels us to consider not the total lost radiation, but rather the radiation scattering out of the NA. So we account for this by considering the radiation scattered such that it is not collected at the NA of the detector as

2 $${Q}_{{{{{{{\rm{sca}}}}}}}}^{{{{{{{{\rm{NA}}}}}}}}}=\frac{1}{{(2\pi \tilde{\nu }a)}^{2}}\int\nolimits_{{\theta }_{{{{{{{{\rm{NA}}}}}}}}}}^{\pi }(| {{{{{{{{\mathcal{S}}}}}}}}}_{1}{| }^{2}+| {{{{{{{{\mathcal{S}}}}}}}}}_{2}{| }^{2})\sin (\theta )d\theta$$

The size of the detector and the distance to the sample determines the numerical aperture *θ*_*NA*_. Simulating the collection over the NA is crucial for making the simulations similar to measured IR spectra. We also considered accounting for the focusing optics in our simulations as well, but found that this didn’t improve our results significantly and since it makes the simulations slower we opted for not including focusing optics in our simulations. We now have the total lost radiation at the numerical aperture of the detector as ${Q}_{{{\mbox{ext}}}}^{NA}={{{{{{{\mathcal{C}}}}}}}}{Q}_{{{\mbox{abs}}}}+{Q}_{{{\mbox{sca}}}}^{{{{{{{{\rm{NA}}}}}}}}}$, where the parameter ${{{{{{{\mathcal{C}}}}}}}}$ determines the ratio of absorption to scattering in the measured spectrum. This gives the absorbance as $A=-\log (1-\frac{g}{G}{Q}_{{{\mbox{ext}}}}^{{{\mbox{NA}}}})$ where *g* is the cross section of the sample and *G* is the size of the detector. This is what the IR spectrometer measures. Finally, we note that $\alpha (\tilde{\nu })$ is related to the absorbance *A* for scatter-free spectra by the Beer–Lambert law which states that in that case, the absorbance is linear to the molecular absorption, concentration of the absorbing species and optical path length. We can then find the complex refractive index from the molecular absorption $\alpha (\tilde{\nu })$ through Kramer’s–Kronig relation as

3 $$\Im \{n(\tilde{\nu })\}=\frac{\ln (10)}{4\pi \tilde{\nu }d}\alpha (\tilde{\nu })$$

4 $$\Re \{n(\tilde{\nu })\}={n}_{0}+\frac{c}{\pi }{{{{{{{\mathcal{P}}}}}}}}\int\nolimits_{0}^{\infty }\frac{\alpha ({{\Omega }})}{{{{\Omega }}}^{2}-{\tilde{\nu }}^{2}}d{{\Omega }}$$

where ${{{{{{{\mathcal{P}}}}}}}}$ denotes the Cauchy principal value of the integral and *c* and *d* scaling parameters, relating to the optical path length and *n*_0_ is the constant, real part of the refractive index which we call the effective refractive index. Generally, *n*_0_ is a function of wavelength, but in the infrared region, it is usually considered to be constant.

### Simulating IR spectral data

We implement a framework for simulating the scattering on samples, as described above, where we calculate the scattering amplitudes ${{{{{{{{\mathcal{S}}}}}}}}}_{1/2}(\theta ,\phi )$ and ultimately the loss of radiation measured at the detector for different distributions of complex refractive index in the concentrically spherical samples.

There are numerically stable algorithms which can calculate the scattering amplitude matrices on concentric spheres, and we implement the scheme as described in ref. ^35^ and can thus seamlessly simulate the scattering on multilayer concentric spheres. We let the chemical absorption of the different layers of the sphere vary, that is we allow the shell and the center of the sphere to be chemically distinct. To simulate the chemical absorption we decompose several sets of IR scatter-free spectra by finding their principal component loadings, which we then can use to sample from the latent spaces defined by the sets of spectra. We used ~1900 spectra from 12 different data sets, made different combinations of these spectra and created about 180 different PCA latent spaces. The spectra were interpolated to a common wavenumber range with 737 spectral channels between 3844 and 1006 cm^−1^.

These sets of spectra used in the different decompositions include absorption spectra of several species of fungi, algae and pollen, lung biopsy spectra and spectra of pure agar, amylose, several lipids, chitin, glucan, gluten, etc. These spectra are decomposed in several different combinations, and we have a total of approximately 180 chemically different latent spaces to sample the molecular absorption from.

We simulate scatter-distorted spectra for a large span of radii *a* ∈ [1.25 and 20 μm] and effective refractive indices *n*_0_ ∈ [1.3, 1.6], and since simulated molecular absorption spectra represent a very wide range of chemical signals this ensures that the model will be applicable to a large amount of measured IR spectra.

We also augment our spectra with some other effects often found in measured IR spectra to make the model generalize well to measured spectra, such as atmospheric CO_2_-peaks in the inactive region where biological samples do not absorb ($\tilde{\nu } \sim 2200-2500\,{{{\mbox{cm}}}}^{-1}$), Gaussian noise, some baseline effects and some small local perturbation of the radii for different wavenumbers and smoothing to suppress ripples in the spectra, since ripples are rarely seen in measured spectra, but are generally present in simulations of perfectly concentrically spherical samples. We also use slightly varying numerical apertures in our simulations since we wish our model to work for spectra from different spectrometers, and we also add some small constant imaginary part to the refractive index representing diffuse attenuation of light traveling through the sample. We can now realistically simulate arbitrarily many measured absorbance spectra, which are used for training the model.

### Deep convolutional neural network

The idea underpinning our model and its architecture is seen in Fig. 1. We employ a model consisting of three parts for 3D IR volumetric imaging. Firstly, an Inverse Scatter Encoder, a 1D DCNN solves the ISP and extracts the chemical information about absorption from molecular groups as well as the physical properties of the system, i.e., the effective refractive index and the morphology. This part of the model starts with several blocks of attention-augmented convolutions (aCNN)^36^, batch-normalization (BN) and pooling layers, and finally a fully-connected layer which decomposes the measured spectrum into the latent space.

We used a convolutional network as such models have been shown to successfully separate physical and chemical information in measured IR spectra through the sequential application of local convolutions^12,13^. However, we also include an attention mechanism^37^ to our CNN in order to explicitly include global spectral information in our model, which we expect to be useful for extracting the scattering features which are present over the whole spectral domain. We found that when the attention mechanism is not included in the model architecture, its performance significantly diminished.

Secondly, we employ a ShapeNet—a 3D DCNN which is trained to represent the distribution of the effective refractive indices in 3D space—and two 1D DCNNs which predict the molecular absorption of the two distinct regions in the sample.

The ShapeNet consists of several 3D transpose convolutional layers, which infer the underlying morphology and the distribution of the effective refractive index, whereas the two networks predicting molecular absorption consist of aCNN, BN and unpooling layers which rebuild the molecular absorption spectra of the cell interior and the cell wall.

The model is trained in two phases, firstly we train the Inverse Scatter Encoder to be able to decompose measured spectra and to predict the radius, cell wall thickness and the real, constant parts of the refractive index of the samples, as well as the pure spectra. That is, we start with a simpler 1D CNN instead of the ShapeNet, where we let the model infer the optical properties in their parameterized form instead of the full 3D distribution, due to computational efficiency.

Thereafter, we use the same model but swap the parameter prediction part of the network with the ShapeNet which predicts the 3D distribution of optical parameters. The ShapeNet has been pre-trained as a 3D convolutional Autoencoder to encode 3D concentric spheres, where the decoder of the trained Autoencoder is extracted as the ShapeNet, which significantly speeds up the training of the ShapeNet. We represent the morphological and optical properties of the samples as three-dimensional matrices, where each voxel’s value represents the value of the refractive index at that point, where we set the refractive index around the sample to be zero to clearly delineate the morphology of the sample from the surroundings. The reason for using the full ShapeNet and not merely predicting the simple parameters of the samples is that the former method generalizes easily to samples which cannot be parameterized as easily. So, it is a proof-of-concept which demonstrates that we can infer the three-dimensional distribution of the complex refractive index.

As a cost function for the prediction of molecular absorption, we use a combination of the mean squared error (MSE) and the Pearson correlation coefficient. The MSE cost function forces the absolute error to be minimized, while the Pearson coefficient minimizes the correlation between the two signals. As for spectral IR microscopic absorbance data, the relative peak heights are the most relevant type of information, the Pearson correlation coefficient is relevant since it is invariant to changes in scale. In addition, we use the MSE cost function to be able to compare the relative overall size of absorbance in the cell wall and cell interior. Furthermore, we found that the MSE penalizes individual peak height anomalies stronger than the Pearson correlation, while the Pearson correlation focus more on the overall spectral shape. Therefore, we applied both of the two metrics as our cost functions.

For the optical parameter prediction, we use MSE weighted to give equal importance to the radii and the effective refractive indices. For the ShapeNet we use the Hausdorff distance *D*(*X*, *Y*)^38^ as the cost function, where *D*(*X*, *Y*) is a metric quantifying the distance between two subsets of a metric space. It can be defined for two non-empty subsets *X* and *Y* of some metric space (*M*, *d*) as

5 $$D(X,Y)=\max \left\{\mathop{\sup }\limits_{x\in X}\mathop{\inf }\limits_{y\in Y}d(x,y),\mathop{\sup }\limits_{y\in Y}\mathop{\inf }\limits_{x\in X}d(x,y)\right\}$$

where the metric space in this case is the ${{\mathbb{R}}}^{3}$ and the Euclidean distance is the metric *d*(*x*, *y*).

### Spectral data for validation

Validation of the models is done on five different data sets containing either FPA hyperspectral images or single-element spectra of two types of pollen, fungal and algal cells, and PMMA samples.

Plant pollen samples included four oak (*Quercus*) species (*Q. robur*, *Q. palustris*, *Q. suber*, and *Q. rotundifolia*), and one juniper (*Juniperus*) species (*J. chinensis*); detailed information on sampling and chemistry of the samples was reported previously^39,40^. Filamentous fungus Mucor circinelloides VI04473 was obtained from the Veterinary Institute (Norwegian University of Life Sciences, Ås, Norway) and grown in variable growth conditions, as reported previously^41–43^. Microalgae *Vischeria polyphem* was obtained from the Culture Collection of Algae at Göttingen University (SAG 38.84), and was cultivated in a flask at 20 ^∘^C, 150 rpm, under fluorescent bulbs with light intensity 50*μ*mol of photons m^−2^s^−1^ in BG-11 (PhytoTech USA) medium. Polymethyl methacrylate (PMMA) microspheres, with a nominal radius of 2.75 μm, were purchased from Microspheres-Nanospheres (Corpuscular Inc, NY), and used without further modifications.

Microscopic transmission measurements of pollen and algal samples were performed by measuring the samples on 1 mm thick zinc selenide (ZnSe) windows, by using a Vertex 70 FTIR spectrometer with a Hyperion 3000 IR microscope (Bruker Optik, Ettlingen, Germany), equipped with a globar mid-IR source and 15× objective. The OPUS 8.2 software (Bruker Optik GmbH, Germany) was used for data acquisition and instrument control, background (reference) spectra were recorded immediately before starting each measurement using the sample-free setup, and visible images of the measured samples were obtained by a charge-coupled device (CCD) camera coupled to the microscope. Algal, fungal and juniper pollen samples were measured with 128 × 128 mercury cadmium telluride (MCT) focal plane array (FPA) liquid nitrogen-cooled detector using a fully open aperture, and were recorded with a total of 128 or 256 scans in the 3850 − 900 cm^−1^ spectral range, with a spectral resolution of 8 cm^−1^, and digital spacing of 3.851 cm^−1^. Oak pollen samples were measured with a single element MCT liquid nitrogen-cooled detector using a square aperture of 30 × 30 μm^2^ size, and were recorded with a total of 128 scans in the 7000 − 600 cm^−1^ spectral range, with a spectral resolution of 2 cm^−1^, and digital spacing of 0.964 cm^−1^.

Microscopic transmission measurements of fungal samples were performed by measuring the samples on 1 mm thick zinc sulfide (ZnS) windows, by using a Cary 670 FTIR spectrometer with a Cary 620 IR microscope (Agilent Technologies, Santa Clara CA, USA), equipped with a globar mid-IR source and 2× objective. The Resolutions Pro software (Agilent, Santa Clara CA, USA) was used for data acquisition and instrument control, background (reference) spectra were recorded immediately before starting each measurement using the sample-free setup, and visible images of the measured samples were obtained by a CCD camera coupled to the microscope. The samples were measured with 128 × 128 mercury cadmium telluride (MCT) focal plane array (FPA) liquid nitrogen-cooled detector using a fully open aperture, and were recorded with a total of 128 scans over the 3950 − 850 cm^−1^ spectral range, with a spectral resolution of 4 cm^−1^, and digital spacing of 1.928 cm^−1^.

The HTS-FTIR measurements of glyceryl trioleate and extracted cell wall from *Mucor circinelloides* were performed using the high throughput screening extension (HTS-XT) unit coupled to the Vertex 70 FTIR spectrometer (both Bruker Optik, Ettlingen, Germany). The OPUS software (Bruker Optik GmbH, Ettlingen, Germany) was used for data acquisition and instrument control, spectra were recorded as the ratio of the sample spectrum to the spectrum of the empty IR transparent microplate, and each sample was measured in triplicate. A total of 10 μL of glyceryl trioleate or homogenized fungal biomass was pipetted onto an IR transparent 384-well silica microplate, and dried at room temperature for 2 h. The HTS-FTIR spectra were recorded with an aperture of 5 mm, with a total of 64 scans over the range of 4000 − 400 cm^−1^, a spectral resolution of 6 cm^−1^, and digital spacing of 1.928 cm^−1^.

Microscopic transmission measurements of PMMA sample were performed by measuring the samples on 1-mm thick barium fluoride (BaF2) windows, by using synchrotron radiation at the SOLEIL synchrotron facility coupled to a Nicolet 5700 FTIR spectrometer with a Nicolet Continuum XL IR microscope (Thermo Scientific, CA, USA), equipped with a 32 × objective, single element MCT liquid nitrogen-cooled detector using a square aperture of 10 × 10μm^2^ size, and were recorded with a total of 128 scans in the 8000 − 650 cm^−1^ spectral range, with a spectral resolution of 4 cm^−1^, and digital spacing of 1.929 cm^−1^. The measurements were conducted at the SMIS infrared beamline as described previously^44^. The OMNIC 8.1 software (Thermo Scientific, CA, USA) was used for data acquisition and instrument control, background (reference) spectra were recorded immediately before starting each measurement using the sample-free setup, and visible images of the measured samples were obtained by a charge-coupled device (CCD) camera coupled to the microscope.

**Fig. 6:**
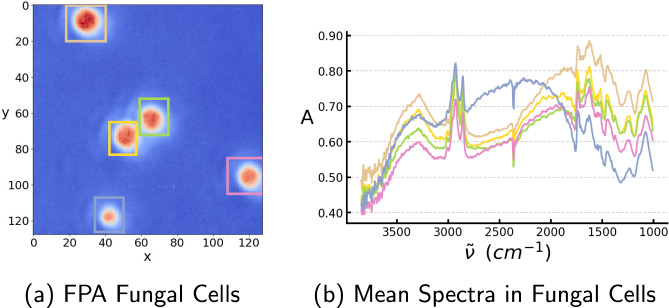
FPA spectral data of filamentous fungi. **a** The FPA image of several *Mucor circinelloides* fungal cells with the fungal cell under consideration in the colored squares. The hyperspectral image is shown for $\tilde{\nu }=1745\,{{{\mbox{cm}}}}^{-1}$. **b** The mean spectra of five fungal cells in the FPA image. The colors of the squares in (**a**) correspond to the colors of the spectra in (**b**).

We use binning to get one spectrum per sample for the samples which have been measured with an FPA imaging system, that is we use the mean spectrum over all the pixels covering our sample of interest in the image as seen in Fig. 6] for the fungal samples. We assume that this is reasonably similar to a single-element measurement, i.e., that not too much radiation is scattered from other samples on the slide into the region of the objective beneath the sample of interest. We see from Fig. 6 that this does indeed seem reasonable, since the average distance between samples is much larger than the average size of the samples. Binning is done such that we can get one spectrum per sample, which is possible to simulate exactly, contrary to the situation with 2D imaging data.

### Optimizing non-learned parameters

There are other unknown parameters for measured spectra, which are not predicted by the model, most notably the scaling parameter in the Kramers–Kronig relations, the ratio ${{{{{{{\mathcal{C}}}}}}}}$ between ${Q}_{sca}^{NA}$ and *Q*_abs_ and baseline, linear, and quadratic effects. Therefore, we use the derivative-free optimization scheme BOBYQA^45^ to determine these free parameters such that the reconstructed spectra fit the measured spectra as well as possible. We note that there is no possible set of free parameters which can, through optimization, yield good reconstructed spectra for incorrectly predicted radii and refractive indices. We verify this by setting the radii and refractive indices randomly and optimizing for the free parameters and confirm that the reconstruction, in this case, does not work very well. The Pearson correlation between measured and predicted parameters from reconstructed spectra were on average *r* = 0.97, and when we randomly selected the parameters it dropped to *r* = 0.92. If we also randomly select the molecular absorption spectra the Pearson correlation drops to about *r* = 0.88.

### Reporting summary

Further information on research design is available in the Nature Portfolio Reporting Summary linked to this article.

## Supplementary information

Supplementary Material

Reporting Summary

## Data Availability

The data supporting the findings of this study could be made available upon reasonable request to the corresponding author via e-mail. Some of the main data used has been archived at 10.5281/zenodo.7386709.

## Code availability

The code developed in this work is available upon request to the corresponding author via e-mail. The main codebase used to develop and train the models can be found at https://github.com/eirikama/sirisp.
