# Supplementary material for: Deep learning-enabled Inference of 3D molecular absorption distribution of biological cells from IR spectra
Source: Commun Chem. 2022 Dec 22;5:175. doi: 10.1038/s42004-022-00792-3 (PMC9814771; doi:10.1038/s42004-022-00792-3)
Supplement: Supplementary file 1 — Supplementary Material [file 42004_2022_792_MOESM1_ESM.pdf]

3D reconstruction of refractive index for three fungal cells

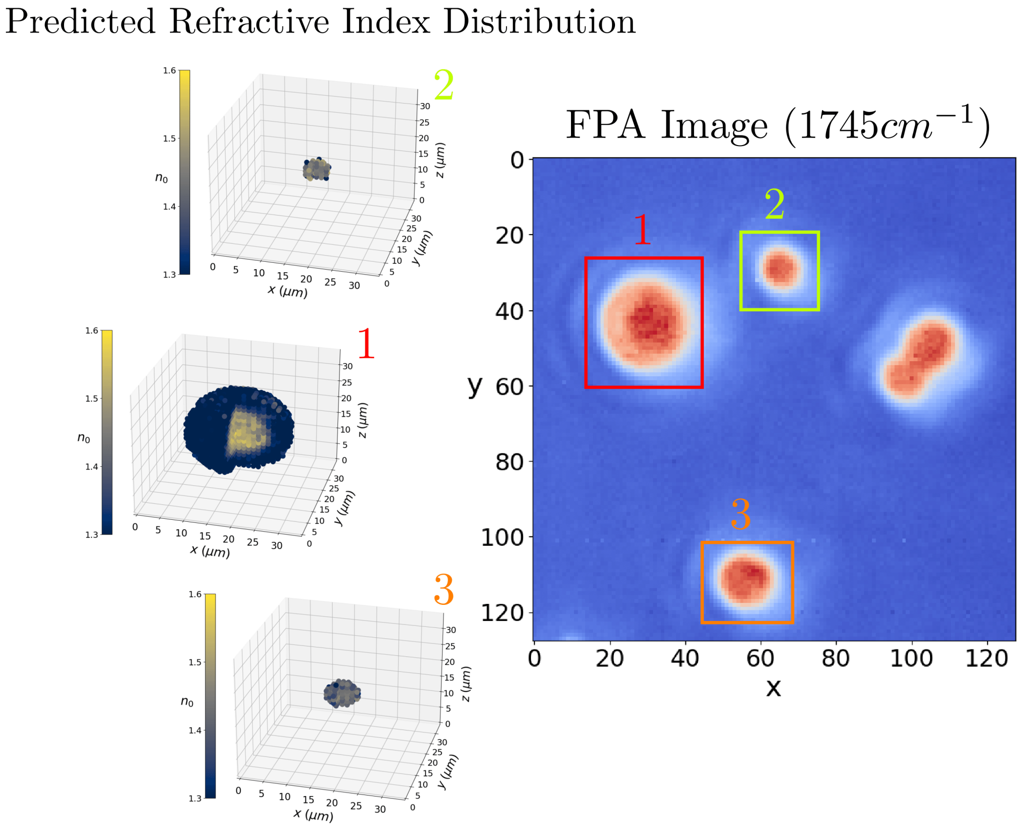

Supplementary Figure 1: *The FPA image at wavenumber $\tilde{\nu} = 1745cm^{-1}$ of several spherical fungal cells. For three fungal cells numbered 1, 2 and 3 above we see the corresponding reconstruction of the 3D distribution of the refractive index by the ShapeNet.*
